# Hyperexpansion of RNA Bacteriophage Diversity

**DOI:** 10.1371/journal.pbio.1002409

**Published:** 2016-03-24

**Authors:** Siddharth R. Krishnamurthy, Andrew B. Janowski, Guoyan Zhao, Dan Barouch, David Wang

**Affiliations:** 1 Departments of Molecular Microbiology and Pathology and Immunology, Washington University School of Medicine, St. Louis, Missouri, United States of America; 2 Center for Virology and Vaccine Research, Beth Israel Deaconess Medical Center, Boston, Massachusetts, United States of America, and Ragon Institute of MGH, MIT, and Harvard, Boston, Massachusetts, United States of America; University of Wisconsin-Madison, UNITED STATES

## Abstract

Bacteriophage modulation of microbial populations impacts critical processes in ocean, soil, and animal ecosystems. However, the role of bacteriophages with RNA genomes (RNA bacteriophages) in these processes is poorly understood, in part because of the limited number of known RNA bacteriophage species. Here, we identify partial genome sequences of 122 RNA bacteriophage phylotypes that are highly divergent from each other and from previously described RNA bacteriophages. These novel RNA bacteriophage sequences were present in samples collected from a range of ecological niches worldwide, including invertebrates and extreme microbial sediment, demonstrating that they are more widely distributed than previously recognized. Genomic analyses of these novel bacteriophages yielded multiple novel genome organizations. Furthermore, one RNA bacteriophage was detected in the transcriptome of a pure culture of *Streptomyces avermitilis*, suggesting for the first time that the known tropism of RNA bacteriophages may include gram-positive bacteria. Finally, reverse transcription PCR (RT-PCR)-based screening for two specific RNA bacteriophages in stool samples from a longitudinal cohort of macaques suggested that they are generally acutely present rather than persistent.

## Abstract 2

This study uses computational metagenomics and molecular experimentation to massively expand the known genomic and ecological diversity of RNA bacteriophages, identifying novel tropisms and genes.

## Author Summary

Bacteriophages (viruses that infect bacteria) can alter biological processes in numerous ecosystems. While there are numerous studies describing the role of bacteriophages with DNA genomes in these processes, the role of bacteriophages with RNA genomes (RNA bacteriophages) is poorly understood. This gap in knowledge is in part because of the limited diversity of known RNA bacteriophages. Here, we begin to address the question by identifying 122 novel RNA bacteriophage partial genome sequences present in metagenomic datasets that are highly divergent from each other and previously described RNA bacteriophages. Additionally, many of these sequences contained novel properties, including novel genes, segmentation, and host range, expanding the frontiers of RNA bacteriophage genomics, evolution, and tropism. These novel RNA bacteriophage sequences were globally distributed from numerous ecological niches, including animal-associated and environmental habitats. These findings will facilitate our understanding of the role of the RNA bacteriophage in microbial communities. Furthermore, there are likely many more unrecognized RNA bacteriophages that remain to be discovered.

## Introduction

Bacteria play key roles in metabolic and immunological processes; however, at this time many of the factors that define the composition of a given microbial population are still unknown [1–4]. Bacteriophages are abundant in many environments, and because they can lyse bacteria or transfer genes, bacteriophages likely play a role in shaping the specific composition of microbial populations. The currently recognized bacteriophages employ highly diverse lifestyles, especially in regards to host range specificity and potential to induce cell lysis, and therefore, bacteriophages from different taxa may uniquely impact the microbial composition of a given niche [5,6]. One particularly understudied area of bacteriophage diversity is that of RNA bacteriophages. While many recent studies have aimed to characterize DNA bacteriophage communities in microbial populations, the RNA bacteriophage component of these communities is poorly defined [7–10].

DNA bacteriophages are currently classified by the International Committee for the Taxonomy of Viruses (ICTV) into eight separate families with a total of 494 species, 55 single-stranded DNA (ssDNA), and 439 double-stranded DNA (dsDNA) bacteriophage species. These species derive from a diverse group of host bacteria; additionally, there are over 1,000 genomic sequences of DNA bacteriophage species in GenBank. By contrast, according to the latest (2014) report of the ICTV, only two official families of RNA bacteriophages are recognized: the single-stranded RNA (ssRNA) bacteriophage family *Leviviridae* that includes four recognized species (Enterobacteria phage Qβ, Enterobacteria phage F1, Enterobacteria phage MS2, and Enterobacteria phage GA) and the segmented, double-stranded RNA (dsRNA) family *Cystoviridae* that contains a single recognized species (Pseudomonas phage ϕ6) [11,12]. There are complete sequences of 11 ssRNA and five dsRNA bacteriophages in the GenBank “Genomes” database as of 20 October 2015, inclusive of the five ICTV-recognized RNA bacteriophage species. In contrast to the DNA bacteriophages, in which bacteriophages have been characterized from a variety of bacterial phyla, all 16 of these bacteriophages are thought to infect hosts within the phylum Proteobacteria, with 15 that infect hosts within the class γ-proteobacteria. In addition, three highly divergent, sewage-derived ssRNA bacteriophage genomes, with unknown host tropisms, were recently deposited in Genbank [13]. For the analyses in this paper, we will refer to these 14 ssRNA bacteriophage sequences and five dsRNA bacteriophages sequences as the “reference RNA bacteriophages.” For some of these RNA bacteriophages, there are additional partial and/or full genomic sequences of closely related variants (share > 66% nucleotide identity to the reference sequences) also available in Genbank.

Bacteriophage identification has historically relied on culture-based methods [14–18]. Given that the majority of bacterial species cannot be cultured in the laboratory, alternative culture-independent methods are necessary to describe bacteriophage diversity [19]. In recent years, metagenomic sequencing has been applied to define bacteriophage populations in the human gut [20–25], skin [26], serum [27], and in the environment [7]. Additionally, computational mining of metagenomic datasets has been valuable for identifying additional novel taxa of DNA bacteriophages [28,29]. However, the vast majority of these studies focused on sequencing and analysis of DNA only and therefore could not evaluate known or novel RNA bacteriophages that may be present. Of the studies that did examine RNA viruses in the environment, only one recent metagenomic study of sewage reported the presence of two novel RNA bacteriophages related to leviviruses [13].

Here, by mining multiple metagenomic datasets that were generated such that RNA could be evaluated, we identify partial genomes of over 120 highly diverse RNA bacteriophage phylotypes that are highly divergent from each other and all of the known RNA bacteriophage genomes. This expansive diversity enabled us to identify new dimensions of RNA bacteriophage biology, including bacteriophages with novel genome organizations, numerous open reading frames (ORFs) that contain novel genes with no detectable homology to known bacteriophage genes, presence in novel ecological niches, and the first data in support of a RNA bacteriophage infection of a gram-positive bacterium. We additionally assess the prevalence of two novel RNA bacteriophages in a cohort of macaques, presenting the first description of the ecological dynamics of these novel RNA bacteriophages. Our results critically illuminate an unexamined dimension of molecular and ecological bacteriophage diversity and fundamentally establish a necessary framework that enables a more accurate dissection of RNA bacteriophage modulation of microbial populations.

## Results and Discussion

### Identification of RNA Bacteriophage Sequences in Local Metagenomic Datasets

To detect RNA bacteriophages, we initially queried multiple metagenomic nucleotide sequence datasets with protein sequences from the known leviviruses and cystoviruses. We focused on datasets generated by our laboratory that contained cDNA sequences derived from RNA in the original material and that represented ecological niches known to support DNA bacteriophages, such as the vertebrate gastrointestinal tract and sewage. Cystovirus protein queries yielded no significant alignments (e-value < 10^−4^). In contrast, multiple nucleotide sequences in datasets from stool-associated and sewage specimens were identified that, following translation to amino acid sequences, aligned to leviviral proteins. The four studies of relevance, which were previously generated by our laboratory, included a study of raw sewage [30], two distinct studies of simian immunodeficiency virus (SIV) infection in nonhuman primates [31,32], and a study of astrovirus infection in mice. Any single dataset that had at least ten sequence reads that yielded significant alignments (e-value < 10^−4^) was selected for assembly. Using a National Center for Biotechnology Information (NCBI) conserved-domain search (e-value < 10^−3^) or Phyre2 (confidence > 90%), partial genomes of RNA bacteriophage phylotypes were defined as any assembled sequence greater than 750 nucleotides in length that contained a translated frame with a recognizable RNA bacteriophage-specific domain, such as a bacteriophage-specific RNA-dependent RNA polymerase (RdRp), capsid, maturation protein, or packaging nucleoside triphosphatase (NTPase) [33,34].

In order to focus our analyses on truly unique RNA bacteriophage phylotypes, any partial genomes that shared > 70% nucleotide identity in either the RdRp or the maturation gene were defined as belonging to a single phylotype. The longest partial genome for a given phylotype was selected as the representative sequence for that phylotype in all downstream analyses. By these criteria, partial genomes of 20 unique RNA bacteriophage phylotypes were identified in 17 distinct specimens. Five partial genomes were assembled from metagenomic data from sewage specimens, 14 were from rhesus macaque stool data, and one was from mouse stool data. Additional partial genomes that shared 85%–97% nucleotide identity to these 20 unique partial genomes were also identified in multiple other specimens in these studies, but they did not represent novel phylotypes by our criteria and therefore were not analyzed further. Based on the sequence diversity of each of these assembled partial genomes within a single phylotype, we believe it is unlikely that these RNA bacteriophages originate from laboratory contamination. The 20 unique bacteriophage phylotypes were sequentially named based on whether it was identified from an environmental or animal specimen, followed by a two-letter descriptor of the ecological niche.

### Experimental Confirmation of Partial Genome Assembly and 3ʹ Termini

To confirm the partial genome assemblies, the eight longest partial genomes (range 3.5–5.0 kb) out of the 20 identified were experimentally validated by generating multiple overlapping reverse transcription PCR (RT-PCR) amplicons followed by Sanger sequencing (S1 Table). The average length of the amplicons was ~1.8 kb; primers used to generate these amplicons are available in S3 Table. In addition, the 3ʹ ends of AVE000, AVE001, and AVE003 were extended using rapid amplification of cDNA ends (RACE).

### RNA Bacteriophages Are Prevalent in Publicly Available Metagenomic Datasets

To expand our search space, we analyzed publicly deposited sequencing datasets—generated by other laboratories—that sequenced RNA (>10,000 Sequence Read Archive [SRA] datasets associated with >2,000 publications). These included transcriptomic and RNA-inclusive metagenomic studies. The metagenomic data analyzed were derived from environmental sources, such as oceans, sewage, and soil, and animal-associated sources, including stool. We aligned amino acid sequences from the 20 novel and 19 reference RNA bacteriophages against sequences in these datasets, following six-frame translation, using tBLASTn. Out of 2,765 RNA-inclusive metagenomes and 7,309 transcriptomic datasets examined, 115 contained at least ten sequences with significant alignments (e-value < 10^−4^). The complete sequencing data from each of these 115 datasets were assembled, and RNA bacteriophage partial genomes were defined as above (length > 750 nt, <70% identity to any other partial or complete genome). We identified 138 unique partial genomes that contained ssRNA bacteriophage domains and five unique partial genomes that contained characteristic dsRNA bacteriophage motifs (S1 and S2 Tables; S1 Dataset). Thus, including the initial identification of the 20 novel ssRNA bacteriophages, we identified a total of 158 unique ssRNA bacteriophage motif-containing partial genomes. For the partial genomes that contained ssRNA bacteriophage-associated domains, 119 contained RdRp domains, and 81 contained maturation domains (42 contained both maturation and RdRp domains). Three partial genomes contained dsRNA bacteriophage RdRp domains. As RNA viruses are not known to encode multiple RdRp genes, we conservatively estimated the number of novel RNA bacteriophage phylotypes based on the number of partial genomes that contain unique RdRp domains. Based on this criterion, we have identified at least 122 novel RNA bacteriophage phylotypes, greatly increasing the known RNA bacteriophage diversity. Furthermore, it is possible that some of the partial genomes that contained only maturation domains may derive from additional novel RNA bacteriophages, so this is likely an underestimation.

### Novel RNA Bacteriophage Genomes Are Highly Divergent from Known Genomes

**Fig 1:**
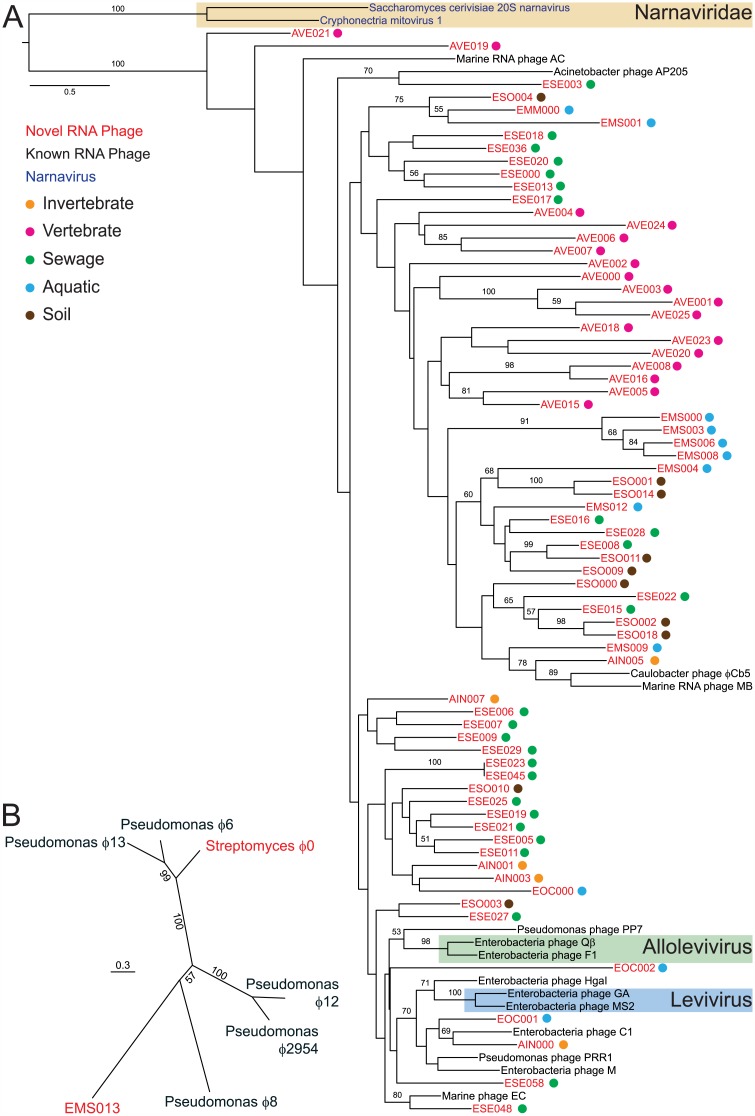
Phylogenetic analyses of novel RNA bacteriophages discovered in metagenomic sequencing datasets. (A) ssRNA RdRp domain-based tree. Colored dots represent the ecological niche from which each partial genome was identified. Bootstrap values under 50 are not displayed. The current ICTV taxonomic classification for RNA bacteriophages and eukaryotic viruses is shaded. (B) Phylogenetic analysis based on RdRp of EMS013 and Streptomyces bacteriophage ϕ0 with known cystoviruses.

To elucidate the evolutionary relationships between the novel and known RNA bacteriophages, we next performed phylogenetic analysis. Of the 119 novel ssRNA RdRp-domain-containing partial genomes, we limited the analysis to the 71 partial genomes that encompassed all five conserved motifs of the RdRp palm domain [35]. In addition, we included the 14 “reference ssRNA bacteriophages.” We included an outgroup containing the RdRp palm domains of the two type species of the family *Narnaviridae* as their polymerases are most closely related to those of leviviruses [36]. While bootstrap support for some portions of the tree is limited, it nonetheless demonstrated that the partial genomes were highly divergent from each other and from the known RNA bacteriophages (Fig 1A). For the dsRNA bacteriophage-domain-containing partial genomes, two unique partial genomes contained the entire RdRp gene, and both were clearly distinct (Fig 1B).

There are currently no official ICTV criteria for defining species among RNA bacteriophages. For many other viral taxa, strictly molecular criteria are used [37,38]. For example, marine picornaviruses have previously been identified by sequence alignment and classified based on phylogenetic distance [38]. Taxonomy of DNA bacteriophages has traditionally relied on bacteriophage morphology but now is largely determined using sequence-based criteria, in that phages that share a certain percentage of genes are considered the same species [39,40]. One possible classification strategy for RNA bacteriophage would be to infer sequence-based criteria based on the current ICTV-recognized species. Distinct ssRNA bacteriophage species within ICTV-recognized genera (levivirus and allolevivirus) share <60% amino acid identity (51% and 55% amino acid identity in the RdRp, respectively). By extension, if membership in a species is defined as sharing ≥60% amino acid identity in the RdRp, the 158 ssRNA RNA bacteriophage phylotypes would represent 111 novel RNA bacteriophage species. Even using a 50% amino acid identity threshold (which would collapse currently recognized distinct species into one species) would still result in 53 novel ssRNA bacteriophage species (Table 1). Regardless of the final criteria used for classification by the ICTV, the RNA bacteriophage phylotypes identified in this study dramatically expand the known sequence diversity of RNA bacteriophages.

**Table 1:** Number of ssRNA bacteriophage species based on varying amino acid identity cut-offs.

Amino acid identity	Number of Species
70	117
60	111
50	53
40	9

The family *Cystoviridae* has a single genus, the only ICTV-recognized species of which is Pseudomonas phage ϕ6 [12]. The four additional fully sequenced dsRNA bacteriophage species in Genbank, which are not officially classified in the genus *Cystovirus*, encode polymerase proteins that share 20%–51% amino acid identity to that of Pseudomonas phage ϕ6. A species defining criterion of 50% amino acid identity would classify the three novel dsRNA bacteriophage phylotypes as three species. A threshold of 40% amino acid identity, which would collapse four distinctly recognized species into two, would result in two novel dsRNA species.

### Multiple Novel Genome Organizations for RNA Bacteriophages Identified

**Fig 2:**
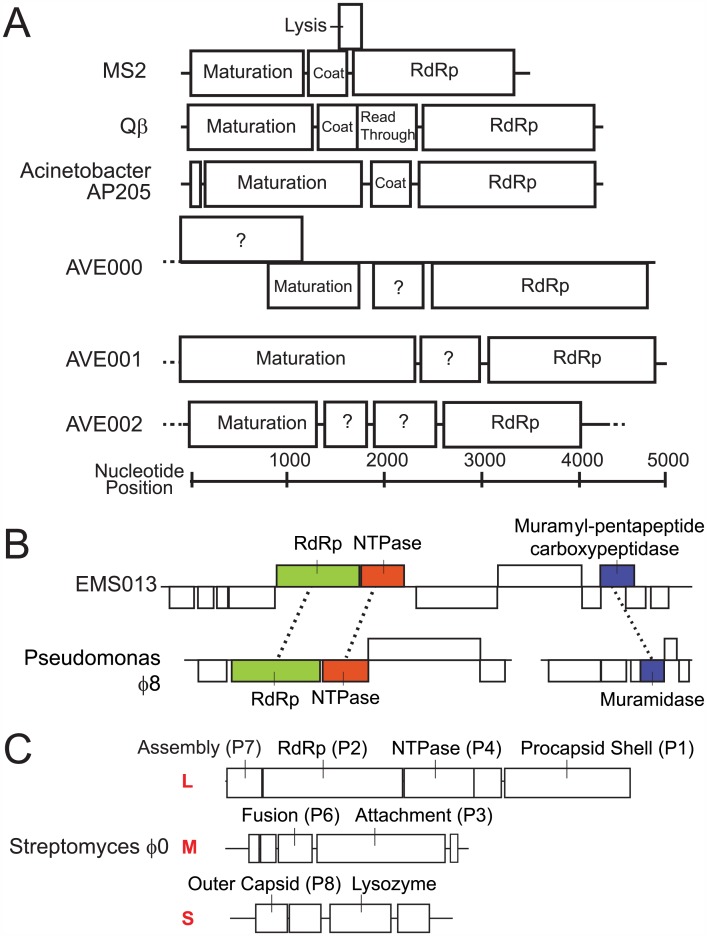
Unique characteristics of novel RNA bacteriophage. (A) Genome organizations of three novel RNA bacteriophage partial genomes compared to prototypical RNA bacteriophage. (B) EMS013 and (C) Streptomyces bacteriophage ϕ0 genome organizations. ORFs were annotated using protein alignment, conserved domain searching, and structural alignment.

Following gene prediction and annotation of all the novel RNA bacteriophages, multiple novel genome organizations were identified. Two of the novel RNA bacteriophage partial genomes, both of which were confirmed by RT-PCR and Sanger sequencing, were much longer than all sequenced leviviruses, which range from 3.73–4.27 kb in length [41]. The genome of AVE000 had at least a 4.95 kb genome; this longer genome can be attributed to the presence of a novel >1.20 kb ORF of unknown function that is 5ʹ to and partially overlaps the maturation protein by 259 nucleotides (Fig 2A). AVE001 also has an expanded genome of at least 5.02 kb, due to the presence of a strikingly large 2.39 kb ORF containing the maturation domain, which is larger than all of the reference ssRNA bacteriophages maturation genes, which on average are 1.27 kb and range from 1.17–1.60 kb. In addition, AVE002 is the first RNA bacteriophage described to contain two nonoverlapping ORFs between the RdRp and maturation genes; neither of the two ORFs has discernable similarity to known proteins. While one of these ORFs likely represents the coat protein, the other ORF might represent a novel lysin or have homologous function to the Qβ read-through protein. From the 119 ssRNA partial genomes, there were 100 ORFs predicted exclusive of the RdRp and maturation genes. Aside from eight ORFs that had predicted leviviral coat domains and one that had a MS2 lysin domain, none of the other 91 ORFs had primary sequence alignment to any known bacteriophage coat or lysin protein. These ORFs may encode proteins that are coat or lysin orthologs that are unrecognizable because of the greater evolutionary divergence of those genes as compared to the RdRp or maturation protein, or they could have completely novel functionalities. Even in the former case, the extreme evolutionary divergence may result in novel host tropisms or novel mechanisms of bacterial lysis. Further elaboration of these bacteriophage genomes will likely identify additional novel genome organizations and additional novel ORFs of unknown function.

The cystovirus protein sequence queries of public sequencing datasets identified partial genomes that provided evidence of an RNA bacteriophage with a novel genomic organization. All five reference cystoviruses are trisegmented, with a ~6.4 kb L segment encoding both the RdRp and packaging NTPase and a ~2.9 kb S segment separately encoding a peptidoglycan degradation enzyme. We identified EMS013, a single 11.2 kb assembled sequence from a metatranscriptomic sample, originally isolated from the Zodletone sulfur spring, containing three individual ORFs that were annotated with these three functions (Fig 2B). Two additional samples from this sulfur spring in the same study also contained sequences that aligned to this partial genome. An assembled sequence that contains both a cystoviral L and S genes is notable as there is ongoing debate as to the evolutionary origin of cystoviruses [42]. One model suggests that cystoviruses share a common eukaryote-infecting ancestor with segmented eukaryotic RNA viruses. The competing model suggests that cystoviruses originate from an unsegmented bacteria-infecting dsRNA bacteriophage. While this partial genome is based solely on in silico assembly, a provocative hypothesis is that this bacteriophage could represent evidence of an unsegmented cystovirus ancestor.

### Identification of a RNA Bacteriophage in a Gram-Positive Bacteria Transcriptome Study

As the majority of the novel RNA bacteriophage partial genomes were detected in metagenomic datasets derived from complex microbial communities, the host bacterium of each bacteriophage could not be explicitly determined by our analyses in most cases. One notable exception was the detection of three dsRNA motif-containing assembled sequences in a publically available bacterial transcriptomic study derived from pure culture of *Streptomyces avermitilis* [43]. This bacteriophage had the traditional genome organization of cystoviruses, although many ORFs could not be definitively annotated by either sequence or structural alignment (Fig 2C). This was named Streptomyces bacteriophage ϕ0, keeping with nomenclature conventions of other cystoviruses. The study was composed of two conditions with three replicates each, and five out of six samples contained sequences from the RNA bacteriophage. The presence of sequences in each of the specimens in this experiment, combined with the annotation of the study as being derived from bacterial monoculture, strongly suggests that *S*. *avermitlis* represents the true host for this bacteriophage. As the known RNA bacteriophages are only believed to infect proteobacteria, Streptomyces bacteriophage ϕ0, if experimentally confirmed to infect *S*. *avermitilis*, would represent the first RNA bacteriophage known to infect bacteria in a phylum other than the proteobacteria. Moreover, it would be the first RNA bacteriophage known to infect a gram-positive bacteria, thereby dramatically broadening the known bacterial host range of RNA bacteriophages.

### RNA Bacteriophages Inhabit Multiple Novel Biogeographies

While many bacteriophages were found from ecologies known to harbor RNA bacteriophages, namely mammalian stool and sewage, we identified numerous bacteriophages from novel ecological niches (S1 Table). Interestingly, we identified numerous bacteriophages that originated from microbial communities of invertebrate hosts, including pools of insects and aquatic invertebrates such as crabs, sponges, and barnacles. Additionally, bacteriophages were identified from microbial sediments associated with extreme aquatic environments, such as sulfur springs and benthic cold seeps.

### RNA Bacteriophages Are Acutely Present in the Microbiome

**Fig 3:**
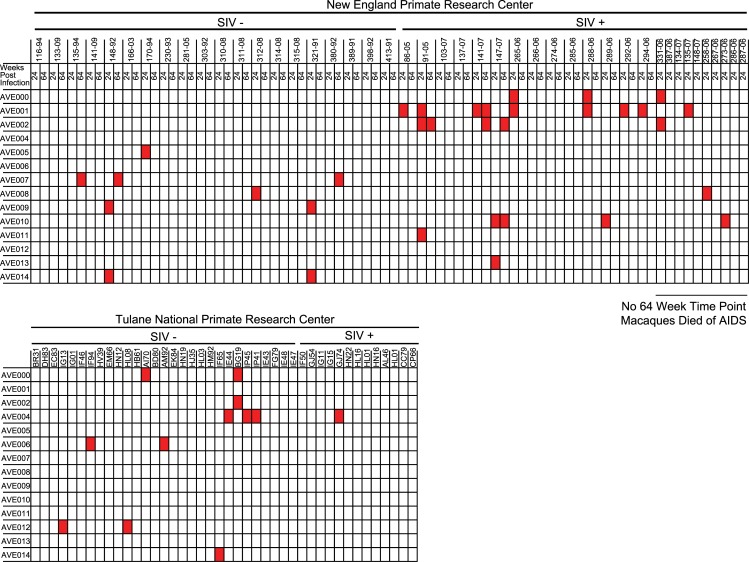
Presence/absence heatmaps of RNA bacteriophage prevalence based on metagenomic sequencing in Rhesus Macaque Study 1.

**Fig 4:**
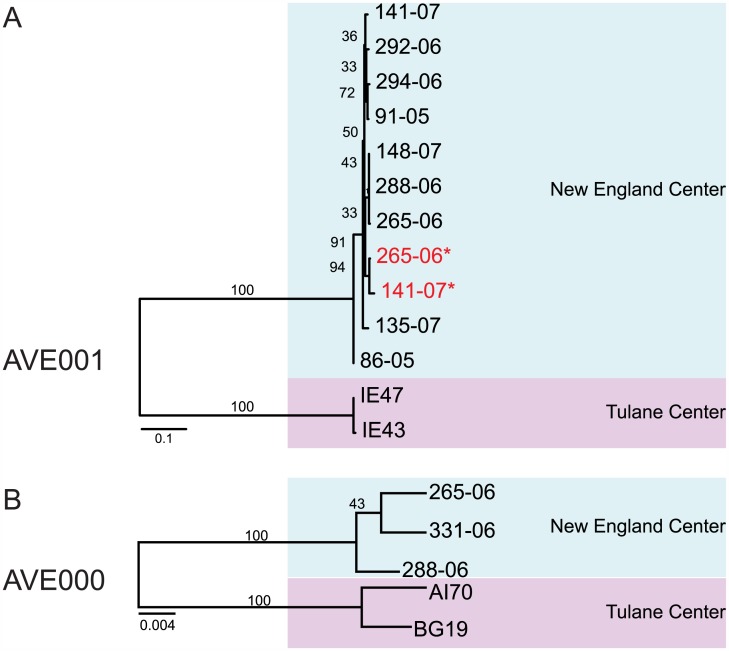
Maximum-likelihood phylogenetic analysis of (A) AVE001- and (B) AVE000-positive amplicons. Red text and asterisks indicate samples from the second time point.

In order to evaluate spatial and temporal trends associated with novel RNA bacteriophages, we defined the prevalence of RNA bacteriophages in stool from previously described cohorts [32] of rhesus macaques collected at the Tulane National Primate Research Center (TNPRC) and the New England Primate Research Center (NEPRC) using both metagenomic and RT-PCR-based approaches. Specimens from NEPRC were available at two separate time points, 24 and 64 wk post SIV infection. Based on metagenomic sequencing data, 34 out of 120 specimens had at least one sequence from one novel RNA bacteriophage, with ten specimens containing at least one sequence from two or more novel RNA bacteriophages (Fig 3). To independently assess the prevalence of a subset of these RNA bacteriophages, we screened this cohort using two sets of PCR primers: one set for AVE000 and another for AVE001. We detected AVE000 in five out of 120 rhesus macaques and detected AVE001 in 13 specimens. The AVE000-positive amplicon sequences shared 95%–99% nucleotide identity with each other, while AVE001-positive amplicons shared 84%–100% nucleotide identity with each other. Phylogenetic analysis demonstrated that there was apparent geographic segregation of both AVE000 and AVE001, as sequences from each primate research center formed distinct clusters (Fig 4, Tables 2 and 3). All specimens positive by metagenomic sequencing for AVE000 or AVE001 were confirmed to be RT-PCR positive. In addition, four specimens that were negative by metagenomic sequencing were RT-PCR positive, most likely because of the increased sensitivity of the RT-PCR assay compared to metagenomic sequencing. The geographic clustering of the amplicon sequences combined with their observed diversity strongly argues against the possibility of laboratory contamination, as all of these specimens were prepared using the same protocol and reagents. From the RT-PCR analysis, we found only two macaques were positive for the same RNA bacteriophage at two separate time points, suggesting that AVE000 and AVE001 generally do not persist (Fig 4). Similarly, from the metagenomic analysis, the vast majority of the RNA bacteriophages were only present at a single time point. This acute presence of these RNA bacteriophages is in stark contrast to the persistent nature in the primate gut of lytic DNA bacteriophages, specifically the *Microviridae* bacteriophages [23].

**Table 2:** Nucleotide alignment identities of AVE001 amplicons from the screening of Rhesus Macaque Study 1. Top half: pairwise nucleotide identity. Bottom half: number of mismatched nucleotides.

Location	Time Point	Specimen	265–06	141–07	135–07	141–07	147–07	265–06	91–05	86–05	294–06	288–06	148–07	IE43	IE47
**NEPRC**	**64**	**265–06**		0.99	0.97	0.98	0.98	0.98	0.97	0.97	0.98	0.98	0.98	0.84	0.84
**NEPRC**	**64**	**141–07**	6		0.97	0.97	0.97	0.97	0.97	0.96	0.97	0.97	0.97	0.84	0.84
**NEPRC**	**24**	**135–07**	12	16		0.98	0.98	0.97	0.98	0.97	0.98	0.97	0.97	0.84	0.85
**NEPRC**	**24**	**141–07**	10	14	11		0.99	0.98	0.99	0.97	0.99	0.99	0.99	0.85	0.85
**NEPRC**	**24**	**147–07**	10	14	11	6		0.98	0.99	0.97	0.99	0.99	0.99	0.84	0.85
**NEPRC**	**24**	**265–06**	10	14	13	8	8		0.97	0.96	0.98	1.00	1.00	0.84	0.84
**NEPRC**	**24**	**91–05**	12	14	11	6	6	12		0.96	0.99	0.98	0.98	0.84	0.85
**NEPRC**	**24**	**86–05**	15	17	14	15	15	17	17		0.97	0.97	0.97	0.86	0.86
**NEPRC**	**24**	**294–06**	11	15	8	5	5	11	3	14		0.98	0.98	0.84	0.85
**NEPRC**	**24**	**288–06**	9	13	12	7	7	1	11	16	10		1.00	0.84	0.85
**NEPRC**	**24**	**148–07**	9	13	12	7	7	1	11	16	10	0		0.84	0.85
**TNPRC**	**NA**	**IE43**	77	78	76	72	76	77	75	67	75	76	76		1.00
**TNPRC**	**NA**	**IE47**	75	76	74	70	74	75	73	65	73	74	74	2	

**Table 3:** Nucleotide alignment identities of AVE000 amplicons from the screening of Rhesus Macaque Study 1. Top half: pairwise nucleotide identity. Bottom half: number of mismatched nucleotides.

Location	Specimen	265–06	288–06	313–08	AI70	BG19
**NEPRC**	**265–06**		0.99	0.99	0.95	0.95
**NEPRC**	**288–06**	5		0.99	0.95	0.95
**NEPRC**	**313–08**	4	5		0.95	0.95
**TNPRC**	**AI70**	23	22	23		0.99
**TNPRC**	**BG19**	20	21	20	5	

In this study, we have vastly increased the number of known RNA bacteriophage phylotypes and demonstrated their presence in a wide range of habitats worldwide. However, this is clearly an underestimate of the total number of RNA bacteriophage species, as there are undoubtedly many more novel RNA bacteriophages that remain undiscovered. While our work has clearly identified a much greater diversity of RNA bacteriophages, there are still obvious taxonomic groups that are missing. For example, RNA bacteriophages that contain negative-sense genomes or helical capsids have still not been identified, both of which underscore some of the many remaining gaps left in our understanding of RNA bacteriophage diversity. Some of these novel RNA bacteriophages may be present in existing metagenomic datasets that simply cannot be currently recognized because of a lack of primary sequence alignment. Furthermore, the vast majority of metagenomic studies are still heavily DNA-centric. With the increased recognition of the importance of RNA bacteriophages and RNA eukaryotic viruses, the number of RNA-inclusive metagenomic datasets will surely grow, leading to additional discoveries of novel RNA bacteriophages. Critically, the multitude of RNA bacteriophages already identified in this study provide a unique opportunity to define their natural contributions to ecology, explore novel aspects of their life cycle, and potentially exploit them as novel tools for bacteriophage therapy.

## Methods

### Ethics Statement

Mouse experiments yielding metagenomic data were performed with the approval of the Washington University IACUC, under protocol #20140244.

### Metagenomic Studies Mined for RNA Bacteriophages

#### Rhesus Macaque Study 1

We analyzed 120 stool specimens that had been previously sequenced using 454 FLX Titanium technology from a previously published study (MG-RAST Project 1449, 1451, 1452) [32]. Briefly, rhesus macaques were either pathogenically infected with SIV or served as controls. They were housed at NEPRC or TNPRC. Macaques at NEPRC were sampled at two time points, 24 and 64 wk post SIV infection, whereas macaques at TNPRC were sampled once.

#### Rhesus Macaque Study 2

From 36 primates that were a subset of a previously published SIV vaccination cohort, 71 stools were collected [31]. Stools were processed as previously described [32]. Stools were diluted 1:6 in PBS, filtered through 0.45 um filter, and extracted on a COBAS Ampliprep Instrument (Roche). Nucleic acids were subjected to random cDNA synthesis and then amplification TruSeq library (Illumina). Libraries were sequenced on an Illumina MiSeq Platform [44]. All sequences were uploaded to the European Nucleotide Archive under the project PRJEB9503.

#### Murine study

Stool pellets combined from two mouse cages infected with murine astrovirus were metagenomically analyzed as previously described [45,46]. Briefly, stool was diluted in 1:6 in PBS and filtered through a 0.45 μm membrane to minimize recovery of intact bacteria. Total nucleic acid was extracted from the filtrate, subjected to random-priming cDNA synthesis and amplification, and sequenced by 454 FLX Titanium pyrosequencing. The sequences from this specimen are deposited in NCBI SRA under PRJNA291303.

#### Sewage study

The sewage sequencing reads have been previously described and are deposited in NCBI SRA (Accession SRA040148) [30]. Briefly, untreated wastewater was obtained from Pittsburgh, Pennsylvania, United States; Barcelona, Spain; and Addis Ababa, Ethiopia, and then virions were concentrated using organic flocculation. Both RNA and DNA were isolated and subsequently sequenced using the 454 FLX Titanium pyrosequencing.

#### tBLASTn query of previously sequenced databases

Nucleotide databases were constructed using either 454 FLX Titanium or Illumina MiSeq sequencing reads from specimens from the above studies. We constructed nucleotide databases from 454 sequencing-based reads that did not align to any known protein within the Genbank non-redundant (nr) database, as determined by VirusHunter [47]. Illumina MiSeq-based nucleotide databases were constructed from sequencing reads that did not align to any known human or eukaryotic viral protein in the non-redundant database, at an e-value of 10^−3^. We downloaded complete reference genomes of all 11 *Leviviridae* and five *Cystoviridae* species, as defined by NCBI Taxonomy, and isolated amino acid sequences of all annotated ORFs. These amino acid sequences were then queried against the described databases using tBLASTn (non-default parameters: -evalue 1e-4 –num_descriptions 100000 –num_alignments 100000 –outfmt 7).

#### tBLASTn query of SRA deposited databases

Using the command line NCBI SRA tBLASTn, amino acid sequences were queried against deposited nucleotide databases that were annotated as having sequenced RNA (S2 Dataset). SRA was searched for datasets using the key terms “stool,” “feces,” “sewage,” “wastewater,” “metatranscriptome,” “ocean,” “viromes,” or “freshwater;” the resulting datasets were further filtered to select those that were annotated to sequence RNA. Similarly, all SRA datasets from individual bacteria were identified by querying the NCBI Taxonomy database with the bacteria taxonomic identification number (taxid2) and then filtering for sequencing of RNA. As these latter studies often contained many specimens, we randomly selected, using a random number generator, a single specimen from each study for analysis; if a specimen had reads that aligned to RNA bacteriophage, every specimen from that study was subsequently analyzed for RNA bacteriophage reads. These datasets were queried using amino acid sequences derived from the 19 reference RNA bacteriophage and 20 novel RNA bacteriophage we identified from initial analysis of our own metagenomic datasets using tBLASTn with the same parameters as above.

#### Partial genome assembly

For every specimen that had more than ten reads that aligned with an e-value < 10^−4^, all deposited reads from the specimen were then assembled with IDBA using default parameters [48]. Specimen datasets larger than 8 GB were split into files smaller than 8 GB for assembly, with each smaller file individually assembled. The contiguous sequences (contigs) resulting from these individually assembled files were then combined into one file and batch assembled together using IDBA. Only contigs longer than 750 nucleotides were selected for further analysis. Because there are no official guidelines from the ICTV for defining a species for RNA bacteriophages, we selected 70% nucleotide identity in the RdRp or maturation gene as a cutoff for defining unique phylotypes in this study, as this cutoff represented the trend from known leviviral species, in which strains can vary as low as 72% nucleotide identity across the RdRp and maturation genes. Therefore, we defined contigs that BLASTn determined to share greater than 70% nucleotide identity across the RdRp or maturation genes as the same phylotype. The longest contig that contained RNA bacteriophage domains from these total assemblies were used as the partial genome.

#### Novel RNA bacteriophage nomenclature

Novel RNA bacteriophages were named based on the ecology from which they were identified. The first letter designates whether the bacteriophage was identified from an animal (A) or an environmental (E) specimen. The second two letters designate a more specific ecological designation, where IN = invertebrate, VE = vertebrate, SE = sewage, SO = soil, MS = microbial sediment, MM = microbial mat, and OC = ocean. The three letters were followed by a three-digit number.

#### Experimental confirmation of contig assembly and 3ʹ termini sequencing

Multiple PCR primers were designed to confirm eight (AVE000, AVE001, AVE002, AVE003, AVE004, AVE005, AVE006, and AVE007) of the assembled partial genomes (S3 Table). RT-PCR was performed using the Qiagen OneStep RT-PCR kit. Gaps between contigs from the same specimen were closed by designing PCR primers from the existing contigs. Using 3ʹ RACE as previously described, 3ʹ termini were confirmed [47]. Briefly, total nucleic acid was polyadenylated (Ambion), column purified (Qiagen RNEasy), and then used as a template in OneStep RT-PCR (Qiagen), using an Oligo-d(T) primer and the primers specified (S3 Table). All amplicons were cloned into pCR4-TOPO and Sanger sequenced. Each partial genome was sequenced to >2x coverage by Sanger sequencing. Discrepancies between next-generation sequencing partial genomes and Sanger sequencing reads were resolved with additional Sanger sequencing.

#### Genomic analysis

ORFs were predicted using ORF Finder from NCBI and subsequently analyzed for conserved domains using the NCBI Conserved-Domain Search. Domain annotation was defined using NCBI conserved domains that had e-values < 10^−3^ or structural alignments with greater than 90% accuracy using Phyre2. Global alignment of RNA bacteriophage proteins was performed with the MUSCLE algorithm using MEGA6, and pairwise percent identity was calculated based on the p-distance based on the alignment matrix.

#### Phylogenetic analysis

ssRNA bacteriophage phylogeny was determined by acquiring RdRp protein sequences from NCBI GenBank for all reference levivirus genomes (Enterobacteria bacteriophage FI: NC_004301.1, Acinetobacter bacteriophage AP205: NC_002700.2, Enterobacteria bacteriophage Qβ: NC_001890.1, Caulobacter bacteriophage ϕCb5: NC_019453.1, Pseudomonas bacteriophage PP7: NC_001628.1, Pseudomonas bacteriophage PRR1: NC_008294.1, Enterobacteria bacteriophage MS2: NC_001417.2, Enterobacteria bacteriophage Hgal1: NC_019922.1, Enterobacteria bacteriophage C-1 INW-2012: NC_019920.1, Enterobacteria bacteriophage GA: NC_001426.1, and Enterobacteria bacteriophage M: NC_019707.1) as well as three additional known ssRNA bacteriophages: Marine bacteriophage MB: KF510034.2, Marine bacteriophage EC: KF616862.2, and Marine bacteriophage AC: KF616864. Additionally, the two type species of the family *Narnaviridae* (Cryphonectria parasitica mitovirus: NC_004046.1, and Saccharomyces cerevisiae narnavirus 20S: NC_004051.1) were included as out-groups. Novel ssRNA bacteriophage that contained all five motifs of the RdRp palm domain as determined by multiple sequence alignment to the known ssRNA bacteriophage were selected for phylogenetic analysis. Amino acid sequences were aligned using 13 iterations of MUSCLE in MEGA6. The palm domain alignment was isolated, and noninformative sequences were trimmed using GBlocks (minimum number of sequences for a conserved position = 20, minimum number of sequences for a flank position = 73, maximum number of contiguous nonconserved positions = 20, minimum length of a block = 4, and allowed gap positions = all) [49]. The final trimmed alignment was manually edited to preserve known conserved RdRp motifs and then used to construct maximum likelihood phylogenetic trees using Blosum62 + F + G + I with 100 bootstrap replicates in PhyML. dsRNA bacteriophage phylogeny was determined by acquiring RdRp protein sequences from NCBI GenBank for all reference cystovirus genomes (Pseudomonas bacteriophage ϕ6: NC_003715.1, Pseudomonas bacteriophage ϕ8: NC_003299.1, Pseudomonas bacteriophage ϕ12: NC_004173.1, Pseudomonas bacteriophage ϕ13: NC_004172.1 and Pseudomonas bacteriophage ϕ2954: NC_012091.2). Novel dsRNA bacteriophages that contained the full RdRp ORF were also selected for phylogenetic analysis. Amino acid sequences were aligned using six iterations of MUSCLE and maximum likelihood phylogenetic trees using LG + F + G + I with 100 bootstrap replicates in MEGA6. Phylogenetic trees that examine single phylotype diversity based on RT-PCR screening were constructed using nucleotide sequence alignments using MUSCLE with six iterations in MEGA6. Maximum likelihood trees were constructed using the Kimura 2-parameter model with 100 bootstraps.

#### RT-PCR screening of samples in Rhesus Macaque Study 1

PCR primers designed to detect AVE000 and AVE001 (S3 Table) were designed. PCR cycling conditions were 50°C for 45 min, 95°C for 15 min, and 40 cycles of 95°C for 30 s, 50°C for 30 s, and 72°C for 1 min, followed by 72°C for 10 min using OneStep RT-PCR (Qiagen).

## Supporting Information

S1 Dataset GenBank entry record for each novel RNA bacteriophage identified from a public dataset. (ZIP)

S2 Dataset List of SRA files searched. (TXT)

S1 Table List of novel ssRNA bacteriophage partial genomes. (XLSX)

S2 Table List of novel dsRNA bacteriophage partial genomes. (XLSX)

S3 Table Primers used for RT-PCR validation of partial genome assemblies and for 3ʹ RACE. (XLSX)

## Abbreviations

dsDNA: double-stranded DNA
dsRNA: double-stranded RNA
ICTV: International Committee for the Taxonomy of Viruses
NCBI: National Center for Biotechnology Information
NEPRC: New England Primate Research Center
NTPase: nucleoside triphosphatase
ORF: open reading frame
RACE: rapid amplification of cDNA ends
RdRp: RNA-dependent RNA polymerase
RT-PCR: reverse transcription PCR
SIV: simian immunodeficiency virus
SRA: Sequence Read Archive
ssDNA: single-stranded DNA
ssRNA: single-stranded RNA
TNPRC: Tulane National Primate Research Center
